# Climate change skepticism and index versus standard crop insurance demand in coastal Bangladesh

**DOI:** 10.1007/s10113-017-1174-9

**Published:** 2017-06-19

**Authors:** Sonia Akter, Timothy J. Krupnik, Fahmida Khanam

**Affiliations:** 1 Lee Kuan Yew School of Public Policy, National University of Singapore, 469C Bukit Timah Road, Singapore 259772, Singapore; 2 Social Sciences Division, International Rice Research Institute, 4031 Los Baños, Laguna, Philippines; 3 International Maize and Wheat Improvement Center (CIMMYT), House 10/B, Road 53, Gulshan-2, Dhaka 1213, Bangladesh

**Keywords:** Weather index insurance, Climate change adaptation, Skepticism, Bangladesh, Choice experiment, Maize

## Abstract

This paper investigates if climate change skepticism, farmers’ fatalistic beliefs, and insurance plan design influence interest in crop weather insurance. While studies of the influence of fatalism on disaster preparedness are common, the ways in which fatalism influences climate change skepticism, and in turn affects farmers’ interest in crop insurance, have not been previously investigated. An additional objective was to understand farmers’ preferences for index versus standard insurance options, the former entailing damage compensation based on post-hazard assessment, the latter tying damage compensation to a set of weather parameter thresholds. A discrete choice experiment was conducted with maize farmers on a climate-risk prone island in coastal Bangladesh. Most farmers were insurance averse. Those who chose insurance were however significantly more likely to select standard as opposed to index-based insurance. Insurance demand was significantly and positively correlated with farmers’ concern about the adverse livelihood impacts of climate change. Farmers who exhibited fatalistic views regarding the consequences of climate change were significantly less likely to opt for insurance of either kind. These findings imply that the prospect for farmers’ investment in insurance is conditioned by their understanding of climate change risks and the utility of adaptation, in addition to insurance scheme design.

## Introduction

Climate change is among the most pressing problems facing future agricultural productivity. Farmers increasingly experience changes in the distribution and occurrence of pests, variable precipitation regimes, thermal stress, and extreme weather hazards (Knutson et al. 2010). Where adaptation measures are not adopted, farmers in South Asia and Sub-Saharan Africa may face considerable production losses (Lobell et al. 2008; Mathew and Akter 2015). These losses may intensify risk perceptions and limit investment in high-value or diversified crop production, thereby creating a poverty trap (Dercon and Christiaensen 2011).

Crop insurance is proposed as a climate change adaptation strategy in developing countries (Binswanger-Mkhize 2012). Despite the potential to reduce risks, farmers’ demand for insurance has however been considerably lower than expected (Akter et al. 2009; Giné et al. 2008). Previous studies identified a range of issues influencing farmers’ low insurance uptake, including financial constraints, unfamiliarity with insurance, low financial literacy, and lack of trust in insurance providers, among others (Akter 2012; Binswanger-Mkhize 2012; Clarke and Grenham 2013; Akter et al. 2016). We further hypothesize that climate change skepticism may be an important impediment to insurance adoption in low-income countries. Climate change “skepticism” refers to the act of rejecting, disputing, or questioning scientific evidence that the global climate is changing, that human actions are responsible for these changes, and that without mitigation and adaptation, serious consequences for humankind may result (Akter et al. 2012; van Rensburg 2015). Literature on perceptions of climate change in developed countries broadly indicates that value orientations, beliefs, identities as well as scientific uncertainty influence mitigation and adaptation practices (Akter and Bennett 2012; Heimann and Mallick 2016). More specific evidence with regards to climate change skepticism suggests that continued public disbelief regarding the trends, causes, and consequences of climate change exerts a strong influence on mitigation and adaptation behavior (Akter et al. 2012; Engels et al. 2013). Climate change skeptics are for example less likely to support mitigation measures such as emissions trading or renewable energy (Akter et al. 2012).

Although widely studied in developed nations (Akter et al. 2012; Engels et al. 2013), climate change skepticism has not been previously investigated among rural people in low-income countries (Heimann and Mallick 2016). Skepticism in a low-income country context may result from many influences including low education, lack of access to information, and perhaps most importantly, from increased prevalence of religious and fatalistic beliefs (Schmuck 2000). Fatalism is a belief framework based on the principle that “everything is preordained,” and is related to a belief in God and their “foreknowledge” in the future. Fatalists maintain worldviews in which many events are understood as caused by “God’s will” (Ringgren 2014). Consequently, some of the potential impacts of climate change may be viewed as being caused by a higher power over which humans have little influence (Ringgren 2014; Misanya and Øyhus 2015). Previous studies focusing on rural Bangladesh’s risk-prone coastal region, for example, indicated that people commonly believe that cyclones are caused by “God’s will”; this belief has been partly responsible for public noncompliance with evacuation orders during several cyclones (Paul 2014). Although evidence to support the prevalence of fatalism and its influence on disaster preparedness is common, the ways in which such convictions induce climate change skepticism, and influence farmers’ preferences for crop weather insurance, are currently nonexistent.

In addition to skepticism, insurance design is likely to play an important role in determining insurance demand. The two most common models of crop weather insurance include (1) standard and (2) index insurance. In standard insurance, payouts for measurable damage or losses are made following crop inspection by an expert. Standard insurance, however, has several limitations. Farmers have been observed purposely and poorly managing their crops to boost the potential of a successful claim, indicating risk of moral hazard (Clarke and Grenham 2013). Standard insurance is also administratively costly; most successful schemes therefore require underwriting by governments, companies, or development organizations. High investment costs also call into question smallholders’ equitable access (Akter et al. 2009; Miranda and Farrin 2012).

Weather index insurance (WII), in which payouts occur when an environmental parameter such as precipitation or temperature surpasses a crop-damaging threshold, has been proposed as an alternative (Binswanger-Mkhize 2012). Theoretically, WII mitigates moral hazards and potentially reduces transaction costs because it does not require posthazard damage assessment. Weather parameter thresholds must be strongly positively correlated with crop losses and reliably measured. Examples include the number of days of successive drought or excessive rain, waterlogging, or extreme temperatures (Clarke and Grenham 2013).

WII however suffers from two common problems, namely basis risk and design complexity. Considering basis risk, indices may be poorly correlated to individual farmers’ experiences. For example, an index may indicate that the threshold for crop damage was not surpassed at a regional level, while specific farmers may nonetheless experience crop losses (Clarke and Grenham 2013). WII is also conceptually complex, disincentivizing smallholders’ willingness to invest (Giné et al. 2008; Akter et al. 2016). This ironically results in “risk aversion,” in which the targeted smallholder clients, especially those in risk-prone regions whom could benefit from climate change adaptation, do not understand the WII products, causing their preference to remain uninsured (Giné et al. 2008).

Funded by a range of international organizations, crop weather insurance projects can be found in Central and South America, Africa, and South and South East Asia (Binswanger-Mkhize 2012; Miranda and Farrin 2012). In some cases, insurance is bundled with credit and savings products to enhance their value and increase demand (Giné and Yang 2009; Stein and Tobacman 2015). Insurance-linked credit shields agricultural loans against weather-related production risks, while insurance-linked savings products are simply insurance contracts with a guaranteed minimum payout. The latter is designed to protect against both idiosyncratic and covariate risks. Yet despite widespread optimism among development practitioners, farmer uptake of both bundled and stand-alone crop WII remains limited (Binswanger-Mkhize 2012). Previous studies indicate that basis risk and design complexity are partly responsible (Binswanger-Mkhize 2012; Akter et al. 2016). Additional questions however remain as to other factors, for example farmers’ preferences for standard insurance, or potential perception of weather events as preordained and hence unavoidable.

This paper examines the ways in which climate change skepticism and product design influence farmers’ demand for crop insurance on a climate change risk-prone island in Bangladesh’s coastal region. Surveys were conducted with 120 recently adopting maize farmers who were first asked a series of questions relating to the three core dimensions of climate change skepticism, namely (1) trend skepticism, considering whether climate change has already started; (2) attribution skepticism, considering whether climate change is caused by human action; and (3) impact skepticism, considering how harmful the impacts of climate change may be without adaptation. Sampled farmers were then presented with a discrete choice experiment (DCE) in which they were asked to choose between different attributes of hypothetical crop insurance contracts. A key attribute examined was the crop damage verification method, which distinguishes between standard and WII. Other DCE attributes included bundling options, risk types, and choice of insurance provider.

### Study region and risk context

As a low-lying deltaic country, Bangladesh is vulnerable to sea level rise, soil and water salinization, as well as extreme weather and cyclones (IPCC 2014). The feasibility of crop weather insurance is consequently being explored as a climate change adaptation instrument by the Government of Bangladesh, the World and Asian Development Banks, research institutes, parastatal insurance corporations, and NGOs (Ahmed and Hasemann 2013).

The island District of Bhola has a long history of cyclones and extreme weather events and was chosen for study. Bhola is located on Bangladesh’s south-central coast, with a population of 1.78 million, 96% of whom are Muslim, with a literacy rate of 43% (BBS 2013). As the staple food, rice production is the core preoccupation of most Bangladeshi farmers. Several development initiatives conversely emphasize crop diversification for income generation. Maize is Bangladesh’s most rapidly expanding cereal, with high profit potential when sold for feedstock into the region’s growing poultry industry (Gathala et al. 2015). Maize has therefore been promoted on Bhola by the USAID and Bill and Melinda Gates Foundation (BMGF)-funded Cereal Systems Initiative for South Asia (CSISA) project, as well as several NGOs, since 2011.

Maize farmers face important climate-related risks. Lobell et al. (2008) used crop and general circulation models to conclude that a 5% maize yield reduction could be expected by 2030 in South Asia. These models however focused primarily on temperature extremes and precipitation variability. Highvelocity winds and hailstorms are also common before the monsoon in coastal environments. These events can cause defoliation and “lodging,” where the crop is knocked down by the wind, breaking stems, injuring roots, and lowering yield. Waterlogging can also result, spurring root degeneration and disease (Timsina et al. 2010). Compared to other lower-yielding cereals or pulses, maize also requires increased seed and nutrient investments, heightening farmers’ risk exposure relative to less capital-intensive crops. Coastal Bangladesh has been impacted by 47 cyclones since 1960 (BBS 2013); cyclone intensity is also expected to increase under climate change (Knutson et al. 2010). Such events can result in serious livelihood impacts and intensify food insecurity (Akter and Mallick 2013; Akter and Basher 2014). Efforts to expand maize cultivation are therefore unlikely to be successful without risk mitigation.

## Materials and methods

### Data collection

The first step of our research design involved preliminary focus group discussions (FGDs) with farmers and agronomists to identify the primary weather-related risks to maize cultivation. We next measured different dimensions of climate change skepticism among farmers. The third step constructed a hypothetical insurance scheme with varying levels of attributes reflecting different product alternatives, followed by a DCE administered to 120 farmers across Bhola Sadar, Borhanuddin, and Daulatkhan subdistricts of Bhola, where maize is most common (Appendix 1 of the Supplementary Materials).

DCEs are a survey-based nonmarket valuation technique, in which we presented respondents with “choice sets” of paired alternative insurance plans (e.g., “plan X,” “plan Y”), simulating an actual marketplace. Each plan consisted of various attribute levels that defined insurance products. The DCE was implemented in October 2014 through farmer interviews conducted by local enumerators. Further details about the survey and data collection procedure are discussed in Appendix 2 of the Supplementary Materials.

### Measuring climate change skepticism

Recent studies have argued that climate change skepticism is a multidimensional concept (Poortinga et al. 2011; Akter et al. 2012), with key dimensions including trend, attribution, and impact skepticism, indicative of disbelief or questioning of the process, causes, or effects of climate change, respectively. Because they are linked to the core assertions of the mainstream climate thesis, these dimensions constitute the core of the climate change skepticism concept (van Rensburg 2015). The three core dimensions (i.e. trend, attribution, and impact) tend to follow a “stepped” pattern or a hierarchy where trend skepticism resides at the top, followed by attribution and impact skepticism (van Rensburg 2015; Akter et al. 2012). A subset of the population who accept trend claims might still reject attribution claims followed by another subset who accept both or either of the trend and attribution claims but are unconvinced about the potential negative impacts of climate change (Akter et al. 2012). Other dimensions of skepticism include process and response skepticism, which are related to the process of generating scientific knowledge and choice of policies, respectively. Each is associated with but not dependent on the core dimensions of skepticism (van Rensburg 2015).

Given the sampled farmers’ low educational level^1^ and limited knowledge of scientific methods and/or climate change mitigation instruments, we focused on the core dimensions of skepticism in this study. Respondents were first asked whether they had heard about climate change and the sources from which they receive such information. They were then asked a simple question to measure trend skepticism: “Have you observed any change in your local climate over the past 20 years?” Respondents were next given a simple, nontechnical description about the trend, cause and projected impacts of global climate change, as based on the IPCC (2014), which documented that the global mean temperature has been rising over the past 100 years, and indicated that this increase is changing climatic patterns in many regions. Respondents were also told that global climate scientists believe the main driver of climate change is excessive greenhouse gas (GHG) pollution, the majority of which results from industrialized nations. Following this statement, care was also taken to assure that respondents understood that while these trends have been observed by scientists, farmers’ and respondents’ own experiences of historical climate patterns could also differ and that both interpretations are equally valid. We therefore reiterated that there is no correct answer to the question of climate change. We finally explained that according to climate scientists’ projections, the future impacts of climate change in Bangladesh might be more severe than the present, with sea level rise and the intensity of natural hazards likely to increase, though we simultaneously stressed that scientists’ and farmers’ impressions of these trends could easily differ.

Following this description, respondents were asked followup questions to measure attribution and impact skepticism: “Do you believe that climate change is caused by harmful pollution emitted by developed countries?” (indicating attribution skepticism); “Do you worry or are you concerned about the harmful impacts of climate change on your lives and livelihoods?” (indicating impact skepticism). Impact skepticism was further measured by examining farmers’ perceptions of future natural hazard risks. They were therefore asked to indicate the perceived probability of future flooding, windstorms, and hailstorms occurring during the drier winter season during which maize is produced.^2^

### Discrete choice experiment design

Six insurance plan attributes were chosen from the preliminary FGDs held with farmers in the study area (Appendix 3 of the Supplementary Materials). These included (1) *Hazard* (*HAZ*), (2) *Verification* (standard vs. index insurance), (3) *Bad Time Payment* (*BTP*) (compensation received by the insured), (4) *Good Time Payment* (*GTP*) (payment to the insured even without damage), (5) *Deposit* (*DEP*) (insurance premium), and (6) *Provider*. The first attribute, *Hazard*, represents the three most common climactic risks reported by maize farmers during FGDs, including (a) excessive and rain inundation, (b) high-velocity winds, and (c) hailstorms.

The *Verification* attributes referred to standard and WII insurance plans. Under standard insurance, crop damage is physically verified, with compensation dependent on the magnitude of assessed damage. The *Bad Time Payment* attribute therefore refers to the maximum indemnity payable by the insurer under a standard insurance contract. For WII, damage assessment is tied to the verification of remotely measured weather parameters, rather than physical assessment. Due to lack of available historical crop damage tied to climactic data, we alternatively worked with experienced agronomists to approximate reasonable weather threshold trigger levels for maize crop damage constructed by combining two weather parameter thresholds for three hazards (Appendix 4 of the Supplementary Materials). Under the WII, the *Bad Time Payment* attribute therefore refers to the indemnity payable by the insurer if the weather parameters cross critical thresholds.

The variable levels of *Good Time Payment* represent insurance bundling options with savings. An attractive feature of a bundled insurance-savings contract is that it offers guaranteed payments regardless of whether outcomes (Stein and Tobacman 2015; Akter et al. 2016). Our hypothetical insurance plans offered three choices to potential clients: *No Return*, and *Partial* and *Full Return.* 3 The *No Return* plan is the stand-alone insurance scheme involving zero *Good Time Payment*. *Partial* and Full Return are partial and full savings within bundled plans, respectively. The No Return plan had relatively lower deposits than *Full* and *Partial Returns.* Interest from deposits into *Full* and *Partial* plans covered insurance premium costs, meaning that the net deposit (i.e., *Deposit − Good Time Payment*) was not substantially different in either plan. Provider was included to identify insurance buyers’ preferred provider for the hypothetical insurance schemes. This attribute addresses concerns raised by research suggesting that low insurance demand partially results from a lack of trust in private providers (Akter et al. 2016; Brouwer and Akter 2010).

**Fig. 1:**
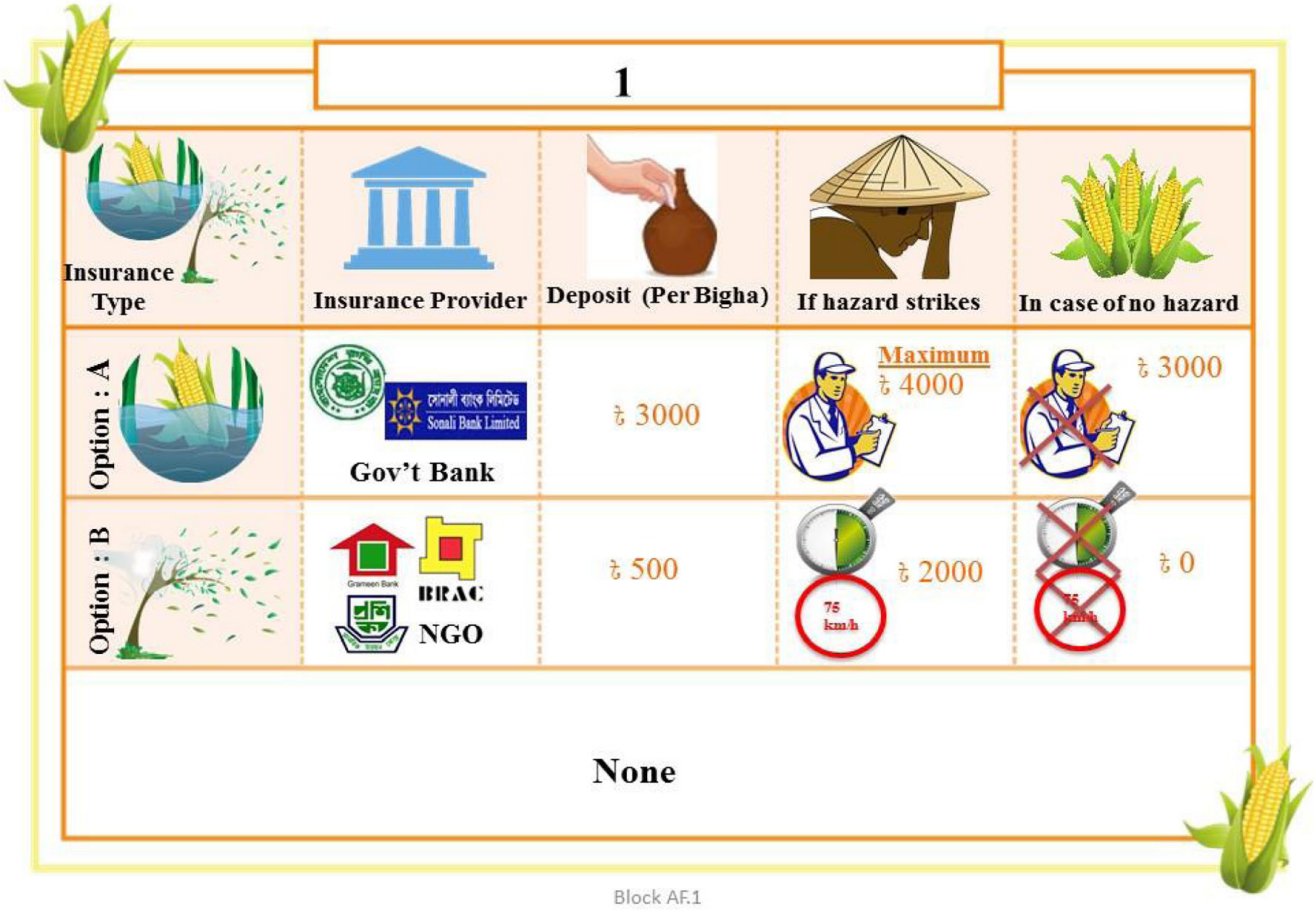
Example of an English-translated choice experiment question depicting options for index insurance type (hail or windstorm crop damage)

Following Bliemer et al. (2008), we constructed a Bayesian efficient DCE design. The DCE included 16 choice combinations randomly divided into four sets. Each respondent therefore answered four unique choice questions, each including two “unlabeled” or “generic” insurance options, plus an opt-out alternative (Fig. 1). A script was read to introduce the hypothetical insurance scheme (Appendix 5 of the Supplementary Materials), after which the choice sets, randomly assigned to the respondents, were presented. The choice questions within each set were also randomized to avoid potential “order bias.”

### Other variables

We collected additional data on respondent’s sociodemographic characteristics, economic status, and assets. Farmers were also asked about incidents of crop damage during the past 10 years. Farmers’ risk preferences were elicited following Eckel and Grossman (2002). The maximum payoff was USD 3.25 (in BDT equivalent), 12.5% less than the daily agricultural wage. Payoffs were determined by coin toss, and the lottery outcome was paid through a telephone-mobile bank account as nominated by each farmer. Time preferences were elicited using a consequential question about whether farmers would prefer to accept their USD 1.30 remuneration for participation in the survey immediately, or as a higher amount (maximum of USD 2.20), after 1 month.

### Analytical model

The random utility model that presents a standard framework for choice experiment results analysis is

$$U_{ij}=V_{ij}\left(X_{ij},\;\beta_{i}\right)+\varepsilon_{ij}\left(X_{ij},\beta_{i}\right)$$ 1

where *V* represents the observable component and *ε* is the unobservable error component of an individual’s utility *i* derived from an alternative *j* (=1, 2, 3). *X_j_* represents choice attributes and *β* denotes a vector of parameters. A concern with such utility frameworks is the potential correlation between the observed (*V_ij_*) and unobserved (*ε_ij_*) components. Unobserved preference heterogeneity embedded in *ε_ij_* thus needs to be explicitly accounted for during the analysis. The random parameter logit (RPL) is a widely used technique that allows for random preference variation (McFadden and Train 2000). In an RPL model, the random parameters *β* are the sum of the population mean, *b*, and a respondent deviation *η_i_* such that

$$U_{ij}=V_{ij}\left(X_{ij}b_{i}+\eta_{i}\right)+\varepsilon_{ij}\left(X_{ij},b_{i}+\eta_{i}\right)$$ 2

where *η_i_* is the stochastic component of the utility which may be correlated among alternatives and across choice sequences. *η_i_* can include normal, lognormal, and triangular distributions.

Using the above framework, the observed component of the indirect utility function of an individual *i* can be specified as:

$$V_{ij}=\beta_{\text{ASC}}*{\text{ASC}}_{ij}+\beta_{\text{DEP}}*{\text{DEP}}_{ij}+\beta_{\text{BTP}}*{\text{BTP}}_{ij}+\beta_{\text{GTP}}*{\text{GTP}}_{ij}+\beta_{\text{Flood}}*{\text{Flood}}_{ij}+\beta_{\text{Wind}}*{\text{Wind}}_{ij}+\beta_{\text{Standard}}*{\text{Standard}}_{ij}+\beta_{\text{Private}}*{\text{Private}}_{ij}$$ 3

where *ASC* is the *Alternative Specific Constant*, 1 for an insurance plan, and zero for the status quo. *ASC* captures the effects of the (nonzero) mean utility associated with the unobserved attributes of each insurance option not explicitly included in the DCE. Two of the hazard types (*Flood* and *Wind*) appear in the model such that the third (*Hail*) is the base category. *Standard* represents the *Verification* attribute such that a standard insurance plan equals 1 and WII is the base category, and *Private* refers to private insurance providers (including private bank and private insurance companies). All other providers such as government banks, NGOs, and Islamic organizations are treated as the base category. In addition to the design features, we hypothesize that the core dimensions of climate change skepticism (i.e., trend, attribution, and impact skepticism) are significant sources of preference heterogeneity for insurance demand. An extended model can therefore be specified to include skepticism indicators and additional insurance design features (i.e., bundling options) and indicators for sociodemographic and economic characteristics:

$$V_{ij}=\beta_{\text{k}}*{\text{ASC}}_{k,ij}+\beta_{\text{DEP}}*{\text{DEP}}_{ij}+\beta_{\text{BTP}}*{\text{BTP}}_{ij}+\beta_{\text{GTP}}*{\text{GTP}}_{ij}+\beta_{\text{Flood}}*{\text{Flood}}_{ij}+\beta_{\text{Wind}}*{\text{Wind}}_{ij}+\beta_{\text{Standard}}*{\text{Standard}}_{ij}+\beta_{\text{Private}}*{\text{Private}}_{ij}+\beta_{\text{s}}(S_{i}^{*}{\text{ASC}}_{k,ij})+\beta_{\text{Z}}({\text{Z}}_{i}^{*}ASC_{k,ij})$$ 4

where *ASC* equals zero for the status quo and 1 for the bundling options (k stands for bundling options *Full, Partial*, and *No Return*), *S* is a vector of skepticism, and *Z* is a vector of variables representing socioeconomic and demographic characteristics. Since the variables in *S* and *Z* are constant for any respondent across choice occasions, they can be included in the utility function as interaction terms with the *ASC* (zero for status quo, 1 for insured).

## Results

### Farmer characteristics

More than half (60%) of the sampled farmers were male (Appendix 6 of the Supplementary Materials). Farmers’ average age was 41, and minimum and maximum age of 20 and 70. Most had very low or no education. Approximately two thirds (63%) had no familiarity with insurance. On average, Fig. 1 Example of an English-translated choice experiment question depicting options for index insurance type (hail or windstorm crop damage) farmers were risk averse as the estimated average risk aversion coefficient (*θ*) was greater than zero (Eckel and Grossman 2002). They were also found to be generally impatient. A majority of respondents (88%) exhibited very high time discount rates. All but 5% were Muslim, and over a third of the households subsisted below the poverty line defined by the BBS (2011). Maize cropped area averaged 0.11 ha.

### Climate change skepticism

Most (85%) of the sampled farmers stated that they had observed climate change over the past 20 years, with the remainder asserting little change (Appendix 7 of the Supplementary Materials). Increasingly heavy monsoon rains, winter season drought, earlier and higher pre-monsoon temperatures, and more frequent flooding were mentioned by farmers as observed changes in the climate. An equal percentage of respondents agreed that climate change has anthropogenic origins. Eleven percent however disagreed, while the remainder stated that they did not know or that only “Allah knows what is true.” Sixty percent of the 11% who rejected the thesis of human contribution to climate change stated their belief that God controls the climate; humans therefore are powerless to induce changes. A small yet nearly significant positive correlation prevailed between belief in climate trends and belief about the causes of climate change (Cramer’s *V* = 0.18, *p* < 0.10); respondents who believed that the climate is changing were therefore slightly more likely to believe in its anthropogenic attribution.

Comparing trend and attribution skepticism, the prevalence of impact skepticism (i.e., lack of concern about impact) was significantly^4^ higher among the quarter of sampled farmers who were unconcerned about the projected harmful impacts of climate change on their lives and livelihoods; rather, they indicated a belief that God would save them from adversities. When respondents’ perceptions about future hazard risks were considered (Appendix 8 of the Supplementary Materials), half of the sampled respondents failed to estimate windstorm probability. More than half (52%) explained that they did not know what the probability of windstorm was and that they believed windstorms are controlled by God. When farmers were asked to state their perceived probability of waterlogging and hail-storms, just over a third stated their perceived frequency, while the rest suggested that they do not know or only God knows as God alone determines the frequencies of these events. Of the respondents who stated a perceived probability of a future hazard occurring, most considered windstorm risks as the most severe. Forty percent believed that extreme windstorms would occur once every year. Hailstorms and inundation were conversely expected to occur once every year by just 21 and 14% of the farmers, respectively.

No significant correlation was observed between trend skepticism and lack of concern about climate change. A low yet significant positive correlation however prevailed between attribution skepticism and lack of concern about climate change impacts (Cramer’s *V* = 0.20, *p* < 0.05). This implies that respondents who believed in the anthropogenic causes of climate change were slightly more likely to be concerned about the projected harmful impacts of climate change.

### Discrete choice experiment results

In Tables 1 and 2, which summarize the estimation results of Eqs. 3 and 4, respectively, all main effect parameters were treated as random. In Table 1, the coefficient of the alternative-specific constant (*β*_ASC_) was negative and statistically significant, implying respondents’ preference to opt out of insurance. The *Deposit* (*β*_DEP_) coefficient was negative and significant, while the coefficients of *Good* and *Bad Time Payment* were positive and significant. These findings conform to a priori theoretical expectations of lower insurance demand due to a higher insurance premium, and higher insurance demand due to higher good and bad time payments, respectively. The estimated standard deviations of *β*_DEP_, *β*_BTP_, and *β*_GTP_ were all highly significant, indicating preference heterogeneity among sampled farmers. The coefficient of *Standard* (*β*_Standard_) was positive and significant, implying that respondents were more likely to choose standard insurance with physical verification over weather index insurance. The standard error of *β*_Standard_ was not significantly different than zero, indicating the absence of significant preference heterogeneity. The coefficient of Private (*β*_Private_) was significant and negative, signifying that respondents were less likely to choose insurance offered by private companies.^5^ No differences in preference heterogeneity were however observed by hazard type, implying that farmer were equally likely to choose coverage against any of the hazard types.

**Table 1:** Random parameter logit regression: main effects model results

Variables	Description	Coefficient (SE)	SD (SE)
Constant parameter			
ASC	Alternative specific constant. Choice of an insured state = 1, otherwise = 0	−0.75** (0.33)	
Random parameters			
* β*_2_ (DEP)^a^	Deposit	−0.0015*** (0.0003)	0.0015*** (0.0003)
* β*_3_ (BTP)^a^	Bad time payment	0.0003*** (0.0001)	0.0003*** (0.0001)
* β*_4_ (GTP)^a^	Good time payment	0.0011*** (0.0002)	0.0011*** (0.0002)
* β*_5_ (Standard)^b^	Standard damage verification process = 1, WII = 0	0.75*** (0.16)	0.60** (0.25)
* β*_6_(Private)^c^	Insurance provider is a private bank or private insurance company = 1, otherwise = 0	−0.67*** (0.24)	0.19(1.30)
* β*_7_(Flood)^d^	Hazard covered by the insurance is flood = 1, otherwise = 0	0.08 (0.24)	0.018 (0.55)
* β*_8_ (Wind)^d^	Hazard covered by the insurance is indstorm = 1, otherwise = 0	0.18(0.23)	0.10 (0.80)
Group number (N)		120	
Log likelihood		−472.67	
LR x^2^		109.33 (*df* =12, *p* < 0.0001)	
McFadden pseudo R^2^		0.10	

*** *p* < 0.01

** *p* < 0.05

*p < 0.10

a Following Hensher and Greene (2003), the coefficients of *Deposit, Bad Time Payment,* and *Good Time Payment* were assigned a bounded triangular distribution in which the location parameter is constrained and equal to its scale. Remaining parameters were assigned a normal distribution.

b Base category = WII (weather index insurance)

c Base category = government banks, NGOs

d Base category = hail

**Table 2:** Random parameter logit regression: extended model to account for conditional heterogeneity

Variables	Description	Coefficient (SE)	SD (SE)
Constant parameters			
Full return	Choice of a full return scheme = 1, otherwise = 0	−2.89* (1.64)	−
Partial return	Choice of a partial return scheme = 1, otherwise = 0	−2.96* (1.60)	−
No return	Choice of a no return scheme = 1, otherwise = 0	−2.64 (1.64)	−
Random parameters			
(*β*_2_ (DEP)^a^	Deposit	−0.001*** (0.0003)	0.001*** (0.0003)
(*β*_3_ (BTP)^a^	Bad time payment	0.0003** (0.0001)	0.0003** (0.0001)
(*β*_4_ (GTP)^a^	Good time payment	0.0009*** (0.0002)	0.0009*** (0.0002)
(*β*_5_ (Standard)^b^	Standard damage verification process = 1, WII = 0	0.78*** (0.16)	0.42 (0.32)
(*β*_6_ (Private)^c^	Insurance provider is a private bank or private insurance company = 1, otherwise = 0	−0.73*** (0.24)	0.24 (0.49)
(*β*_7_ (Flood)^d^	Hazard covered by the insurance is flood = 1, otherwise = 0	0.39 (0.31)	0.04 (0.88)
(*β*_8_ (Wind)^d^	Hazard covered by the insurance is windstorm = 1, otherwise = 0	0.74** (0.31)	0.02 (0.41)
Skepticism indicators			
Attribution	Interaction between ASC and belief that “climate change is caused by human actions”	0.52 (0.42)	−
Trend	Interaction between ASC and belief that “climate is changing”	0.02 (0.41)	−
Impact	Interaction between ASC and those who are concerned about the adverse impacts of climate change	0.88*** (0.34)	−
Flood x P(Flood)	Interaction between flood insurance and perceived positive probability of flood occurring in the future	0.17 (0.38)	−
Wind x P(Wind)	Interaction between wind insurance and perceived positive probability of windstorm occurring in the future	−0.48 (0.37)	−
Hail x P(Hail)	Interaction between hail insurance and perceived positive probability of hailstorm occurring in the future	0.99*** (0.40)	−
Socioeconomic and other attitudinal characteristics				
Risk preference	Interaction between ASC and coefficient of risk preference	−0.01 (0.16)	−
Time preference	Interaction between ASC and discount rate	−0.44 (1.38)	−
Farm size	Interaction between ASC and size of the maize farm	0.01 (0.009)	−
Expenditure	Interaction between ASC and per capita household expenditure	0.0016 (0.96D-04)	−
Asset	Interaction between ASC and value of household asset	0.004*** (0.001)	−
Female	Interaction between ASC and respondent is a female	−0.10 (0.35)	−
Age	Interaction between ASC and respondent's age	0.009 (0.012)	−
Literate	Interaction between ASC and respondent is literate	0.41 (0.36)	−
HH size	Interaction between ASC and household size	−0.17** (0.08)	−
Religion	Interaction between ASC and respondent's (household's) religion is Islam	0.19 (0.70)	−
Familiarity	Interaction between ASC and respondent is familiar with insurance	−0.03 (0.42)	−
Formal savings	Interaction between ASC and household has a formal savings account	0.46 (0.32)	−
Formal credit	Interaction between ASC and household has access to formal credit	0.29 (0.29)	−
Insurance	Interaction between ASC and household purchased insurance before	0.03 (0.48)	–
Daulatkhan district	Interaction between ASC and Daulatkhan district	−0.57 (0.37)	–
Burhanuddin district	Interaction between ASC and Burhanuddin district	−0.34 (0.37)	–
Model fit statistics			
Group number *(N)*		120	
Log likelihood		−445.67	
LR χ^2^		163.31 (*df*=36,*p* < 0.0001)	
McFadden pseudo *R^2^*		0.15	

*** *p* < 0.01

** *p* < 0.05

*p < 0.10

a Following Hensher and Greene (2003), the coefficients of *Deposit, Bad Time Payment,* and *Good Time Payment* were assigned a bounded triangular distribution in which the location parameter is constrained and equal to its scale. Remaining parameters were assigned a normal distribution.

b Base category = WII (weather index insurance)

c Base category = government banks, NGOs

d Base category = hail

In Table 2, which presents the estimation results of Eq. 4, the mean utility coefficients of all bundling options, No Return, Partial Return, and Full Return, were negative. Two were statistically significant at the 10% level. These coefficients were however not significantly different from each other, indicating that, contrary to our hypothesis that an insurance scheme bundled with savings would be more popular, farmers’ decisions to opt for insurance did not vary significantly across bundling options. The signs and significance of most main effect coefficients remained the same as in Table 1 except for the coefficient for *Wind* which was significant at the 5% level, implying significantly higher demand for insurance against windstorms compared to hailstorms.

Two of the five indicators of climate change skepticism, concern that climate change will impact farmers’ livelihoods (*Impact*) and the interaction between Hail and perceived probability of hailstorm occurring in the future (*Hail × P(Hail)*), were also significant. The mean coefficient for both was positive, implying that maize farmers who were concerned about the negative impacts of climate change were significantly more likely to opt for insurance. Similarly, farmers who perceived the risk of experiencing maize damage from future hailstorms, as opposed to those who failed to estimate a probability of hailstorm or those who believed that God determines hailstorm frequency, were significantly more likely to opt for insurance. Perceptions of wind and inundation risk however did not have any significant impact on insurance demand. As expected, poverty (measured in terms of household wealth) significantly influenced insurance choice. Relatively well-off households with a higher asset profile were significantly more likely to choose insurance, although larger households were not, which may be indicative of the need to spread household income across a larger number of family members, thereby lowering interest in insurance investment. Neither individual risk nor time preference, nor spatial heterogeneity were significant determinants of insurance choice. Other respondent characteristics (e.g., demographics, familiarity with insurance, access to formal savings and credit, familiarity with insurance) were insignificant determinants of preference heterogeneity.

### Marginal willingness to pay estimates

The general formula for marginal willingness to pay (MWTP) (or implicit price) of an attribute from a DCE is

MWTP=−(βxβy) 5

where *β_x_* is the coefficient of attribute *x* and *β_y_* is the coefficient of the variable representing payment, in this case *Deposit*. Using the parameter estimates for Table 1 (Eq. 3), we estimated MWTP for each attribute of the hypothetical insurance contract for 33 decimal (0.13 ha) of maize cropped area (Appendix 9 of the Supplementary Materials). The MWTP for *Bad Time Payment* refers to the mean MWTP for a stand-alone WII contract provided by government or an NGO offering USD 13 compensation following a hailstorm event. The mean MWTP for a stand-alone *Hailstorm* WII contract was USD 2.80, significantly different than zero. When a *Good Time Payment* or a savings component of USD 13 is added to the contract, the MWTP increased by USD 9.32, also significantly different than zero. The total MWTP for a *Hailstorm*-based WII contract offering USD 13 payment both in good and bad states of the world sums to USD 12.12, which is not significantly different than USD 13, implying that, on average, the sampled farmers were willing to pay roughly USD 13 to receive the same amount in both good and bad states of the world.

The MWTP for standard hailstorm insurance was USD 6.52, significantly different than zero. Respondents were therefore willing to pay USD 6.52 more on average for insurance that offered post-hazard damage verification, as opposed to WII that relies on remote measurement weather parameter thresholds. The estimated MWTP for private providers (i.e., banks and insurance companies) was negative USD 6.64 and significantly different than zero. Respondents were on average willing to pay less for a hailstorm-based WII offered by a private provider rather than by government or an NGO.

Finally, the estimated differences in MWTP for Flood and *Windstorm* coverage compared to *Hailstorm* insurance were not significantly different than zero.

## Discussion and conclusions

Similar to previous studies in rural Bangladesh that report farmers’ experiences of changing climactic conditions (Ahsan and Brandt 2015) — at times with striking correlation between meteorological evidence and farmers’ recollection of short-term climactic histories (Shameem et al. 2015) — 85% of the farmers surveyed in our study reported having some-how observed the trend of climate change during the past decades. The same percentage of the sampled respondents accepted the thesis that climate change has anthropogenic origins. Despite widespread agreement with both the trend and attribution claims of the climate thesis, a significantly lower proportion of the sampled farmers (75%) expressed their concern that climate change may continue to negatively affect their livelihoods in the future. Interestingly, these numbers are similar to the pattern of skepticism observed in developed countries, particularly in terms of trend and impact skepticism. Akter et al. (2012) and Poortinga et al. (2011) found 78% of sampled respondents agreeing with the trend claim, followed by 75% of the respondents in Akter et al. (2012) and 69% in Poortinga et al. (2011) agreeing with the impact claims. The proportion of the sample in agreement with the attribution claim in the Akter et al. (2012) and Poortinga et al. (2011) studies were 72 and 31%, respectively, which are substantially lower than our findings (85%).

Consistent with literature indicating low demand for crop weather insurance products in developing countries, surveyed farmers were generally averse to the idea of maize crop insurance. A lack of concern for future negative livelihood impacts (i.e., impact skepticism) was an important indicator of insurance aversion. Our study also presents preliminary evidence to suggest that the impact skepticism is induced by fatalistic beliefs. However, to what extent respondents’ lack of knowledge about climate change and the operationalization of the measures of impact skepticism influenced such outcomes remains a research gap. Such responses could for example have resulted from a low level of education. An understanding of the probabilistic nature of weather risks has been observed as influential in determining farmers’ climate adaptation behaviors (Osbahr et al. 2011), and as such, future research that integrates efforts to educate farmers targeted for crop insurance programs on probabilistic weather risks may be beneficial. Our preliminary findings nonetheless support the hypothesis that in the absence of such efforts, climate change skepticism can be an important impediment to increased insurance adoption in low-income regions. In addition to South Asia, agricultural insurance has received considerable research and development policy attention in Africa, South East Asia, and South and Central America, in response to myriad climate-related threats including drought, extreme storms, flooding, and pest outbreaks for both crops and livestock (Binswanger-Mkhize 2012; Greatrex et al. 2015). We are however unaware of efforts to explicitly account for cultural influences including skepticism or fatalistic beliefs in research of development programs that address insurance for farmers or pastoralists. Further research efforts to assess if similar constraints are found in different farming systems and cultural environments may therefore be of wider interest to improve insurance program design and rural development interventions.

In terms of insurance design features, our sampled farmers revealed a strong preference for standard insurance with physical verification of crop damage rather than WII, echoing previous observations of smallholders’ concerns related to basis risk and design complexity articulated elsewhere (Binswanger-Mkhize 2012; Akter et al. 2016). The sampled farmers further exhibited a strong preference against private insurance providers, similar to Brouwer and Akter (2010) and Akter et al. (2016), reconfirming that insurance provider type plays a crucial role in determining insurance adoption. Although an insurance contract bundled with a savings component has theoretical appeal, namely that adoption is expected to be greater than with stand-alone insurance, we found no evidence to support this proposition. Among other factors, wealth was as expected a significant determinant of crop weather insurance demand. This finding problematizes the prospect of crop insurance as a safety net for resource-poor farmers in climate coastal risk regions (Akter 2012; Binswanger-Mkhize 2012), highlighting another factor responsible for low insurance demand in developing countries.

As a climate change adaptation strategy, the potential for crop insurance adoption among farmers in coastal Bangladesh therefore appears to rely not only on the factors that have been identified as important by existing insurance studies, such as lack of trust in insurance providers, low financial literacy, poverty, etc., but also on important yet rarely considered issues including farmers’ degree of belief in the anthropogenic causes and consequences of climate change. This is in addition to their perceptions of the importance of adaptation for the maintenance of their livelihoods. In addition to the difficulty that many smallholders may experience in paying insurance premiums (Binswanger-Mkhize 2012), these reasons appear to partially drive the failure of private sector investment in crop insurance schemes and the frequent need to underwrite both pilot and established insurance projects (Miranda and Farrin 2012). Also working in coastal Bangladesh, Ahsan and Brandt (2015) hypothesized that increased involvement of farming communities is crucial in the planning and development of viable climate change adaptation strategies, and in the successful prioritization and implementation of policies to support them. Our findings provide general backing for this thesis. We conclude that as a prerequisite for meaningful climate adaptation programs, governments, donors, banks, and development projects planning crop insurance schemes would benefit from prior investigation into the ways in which different types of smallholder farmers perceive of climate change, and their interest in adapting to it, in order to better target crop protection interventions.

## Footnotes

1 Over a quintal (22%) of the sampled respondents were illiterate. About half of the sampled respondents (49%) went to primary school and about a quintal (19%) went to secondary school. The rest completed secondary school (8%), college (0.8%), and went to university (1.7%).

2 Farmers were asked to indicate how frequently (once in how many years) they expect to experience inundation due to river flooding and excessive rain, windstorm, and hailstorm in the future.

3 For *No Return* (i.e., a stand-alone insurance), plan *Good Time Payment* = 0. For Partial Return, 0 < Good Time Payment < *Deposit*. For *Full Return, Good Time Payment = Deposit*

4 Trend versus concern: Z = 2.20, *p* < 0.05; attribution versus concern: Z = 2, *p* < 0.05.

5 Note that the coefficient of none of the other providers, except for private bank and private insurance companies, was significantly different than zero. Hence, the coefficient of private versus nonprivate providers has been reported here.
