# Supplementary material for: Climate change skepticism and index versus standard crop insurance demand in coastal Bangladesh
Source: Reg Environ Change. 2017 Jun 19;17:2456–66. doi: 10.1007/s10113-017-1174-9 (PMC6988121; doi:10.1007/s10113-017-1174-9)
Supplement: Supplementary file 1 [file REC-2017-s10113-017-1174-9-S1.docx]

**Appendix 1.** Study area and sampled maize farming households.

**
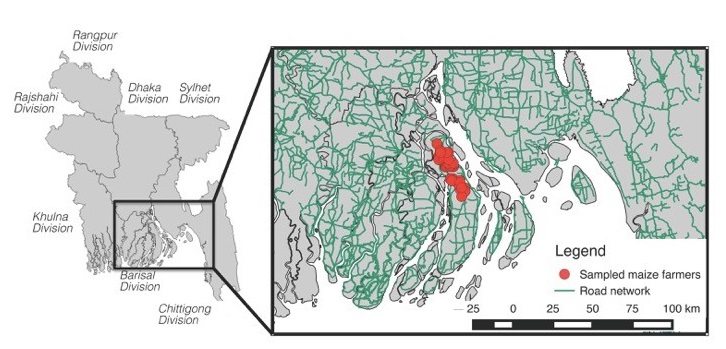
**

**Appendix 2.** Data Collection

- The sample frame included 30 percent women farmers, most of whom lived in male-headed households. The sample were drawn from lists of male and female maize farmers, collected from the Department of Agricultural Extension and CIMMYT. Twenty enumerators, including six women, conducted face-to-face structured interviews. Female enumerators generally interviewed female respondents. Enumerators were full- or part-time students from local colleges having no affiliation to any government or non-governmental organizations.
- Two of the principal investigators provided training in Bangla (4-days) to the enumerators on the survey instruments and how to administer them to the respondents. Trainings involved simulations and pre-tests to assure they were able to correctly administer the surveys in Bangla.
- Interviews with the sampled maize farmers were scheduled by mobile phone for all sampled respondents. They were informed that an enumerator would ask detailed questions about farming practices and costs and returns from maize production. If the respondent, particularly the female respondent, was not fully aware of this information, then the presence of the person in charge of farming was requested.
- All of the women who were sampled participated in the interview. We instructed enumerators to take note of cases where men dominated during the interview by overpowering women and suppressing their voice. However, they reported no such cases. Women were more likely to have a second person present and to consult with them. They included a man in 60% of the interviews and another woman in 23%. When present, the man was almost always consulted during the interview of the choice module. When a woman was present she was consulted in 76% of the interviews. Male respondents were much less likely to have a second person present and it was equally likely that it would be another man or a woman. They were less likely to consult with the second person, doing so in only 76% of the cases in which an additional man was present and in 69% of the cases when a woman was present.

**Appendix 3.** Choice experiment attributes and their associated levels (all monetary values are presented in Bangladesh taka, Tk^a^)

| **Bundling options** | **Attributes** | **Levels** |
| --- | --- | --- |
| No Return | Type | Index, Standard |
|  | Hazard | Flood, Windstorm, Hailstorm |
|  | Deposit^b^ | 100, 200, 300, 500, 800, 1000 |
|  | Guaranteed good time payment | 0 |
|  | Bad time payment | 1000, 1500, 2000, 3000, 5000 |
|  | Provider | Government Bank, NGOs, Private Bank, Private Insurance Companies, Islamic Organizations |
| Partial Return | Type | Index, Standard |
|  | Hazard | Flood, Windstorm, Hailstorm |
|  | Deposit^c^ | 500, 800, 1000, 2000, 2500, 3000 |
|  | Guaranteed good time payment | 200, 800, 1800, 2000, 2500, 2800 |
|  | Bad time payment | 2000, 3000, 4000, 5000 |
|  | Provider | Government Bank, NGOs, Private Bank, Private Insurance Companies, Islamic Organizations |
| Full Return | Type | Index, Standard |
|  | Hazard | Flood, Windstorm, Hailstorm |
|  | Deposit^d^ | 800, 1500, 2000, 2500, 3000, 4000 |
|  | Guaranteed good time payment | 1500, 2000, 2500, 3000, 4000 |
|  | Bad time payment | 1800, 2000, 2500, 3000, 3500, 4000, 5000 |
|  | Provider | Government Bank, NGOs, Private Bank, Private Insurance Companies, Islamic Organizations |

^a^ Tk 77 = 1 USD

^b^ Net deposit (i.e. deposit–good time payment) =100, 200, 300, 500, 800, 1000.

^c^ Net deposit =100, 200, 300, 500, 600.

^d^ Net deposit = 0.

Note: in Bangladesh, the nominal interest rate on a general savings account varies between 6% and 9%

(Bangladesh Bank, 2015).

**Appendix 4.** English translated examples of index insurance trigger flash cards used in the choice experiment, including (A) prolonged crop inundation, (B) hailstorm, and (C) heavy windstorm causing crop lodging.

| (A) 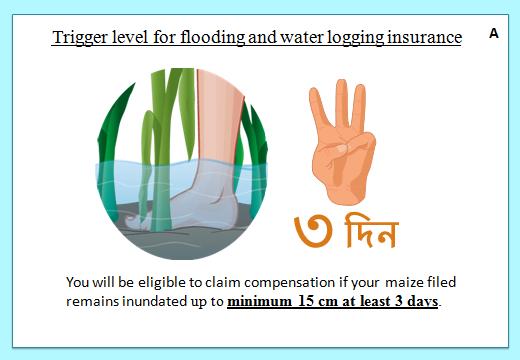 |
| --- |
| (B) 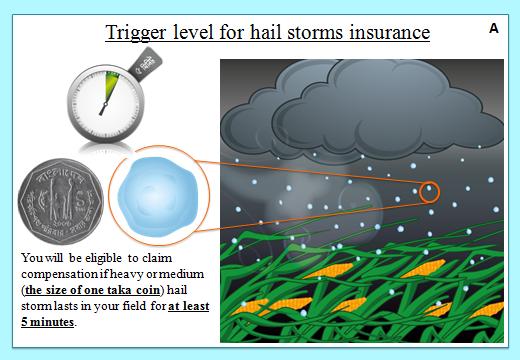 |
| (C)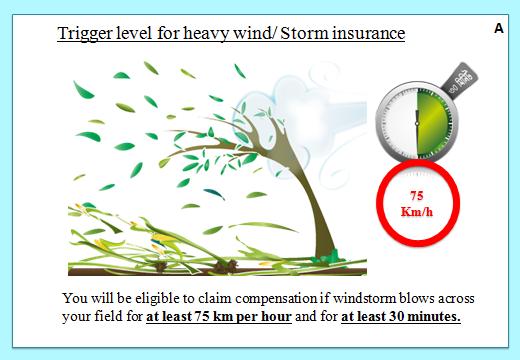 |

Note:

Index insurance trigger levels were designed to reflect hazard intensity and duration. For inundation, the triggers were defined as 15 cm (intensity) and 3 days (duration). Windstorm triggers were set to 75km/h (intensity) and 30 minutes (duration). Hailstorm triggers were 25mm size (intensity) and 5 minutes (duration). To assure farmers understood the hailstorm intensity trigger, hail size circumference was compared to the sphere equivalent of a 1 Bangladesh taka coin.

**Appendix 5.** Description of the insurance scheme presented to survey respondents

The script below is an English translation of the script read to farmer respondents in Bangla to explain the hypothetical index insurance options presented in the discrete choice experiment.

_____________________________________________________

Let me first explain how an insurance scheme works even if you are already familiar with it.

- Insurance is a financial mechanism that helps to reduce risk. If you buy insurance you get compensated for hazard related losses.
- You might have heard about health or life insurance. Health insurance requires you to pay a risk premium periodically (once a year or once every six months). In return you get reimbursed for medical expenses incurred due to certain health problems. Life insurance compensates the insured’s household for sudden or unexpected losses of life of the insured, or for the loss of one/multiple limb/s.
- Similar arrangements can be made to cover crop damage losses arising from weather related hazards such as heavy wind, inundation, and hailstorms. Such insurance schemes are known as ‘weather insurance’.

I will now describe the general principles of a weather insurance scheme for maize for three different hazards:

1. Inundation
2. Windstorm
3. Hailstorm

These three hazards have been identified to be the most serious weather related problems for maize after discussions with agricultural scientists, local agricultural extension workers, and farmers.

If you want to buy the insurance, you will need to:

- Choose a hazard against which you would like to protect your crop.
- Make a deposit in the beginning of the *rabi* (or *boro*) season [the 1^st^ day of *Poush* (December 15)]

In return:

- You will receive a compensation payment on June 15 (the 1^st^ day of *Ashar*) if your chosen hazard takes place.
- If the hazard does not take place, you may or may not receive any money back. This will depend on the type of plan you choose.

Verification:

The payment will be made upon verification of weather indices. There are two methods by which verification can be done: (1) Weather index based and (2) Damage assessment based.

Weather index based verification method:

- Under this method there will be no physical assessment of actual crop damage on your farm. Your compensation will depend on the verification of pre-specified weather thresholds known as a ‘danger level’.
- These insurance schemes have different danger levels. Please take a careful look at the following cards that explain the trigger levels for each of the hazard types.

*Note for the enumerator:*

*Show the cards related to trigger levels one by one and explain them. Ask the respondent if he/she clearly understands them. If he/she does not understand, explain again. Do not proceed until the respondent fully understands the trigger levels.*

- Verification of these weather parameters will be done by experts using local weather stations.
- There is currently no weather station in your village or union. But if weather index insurance is offered in your village in future, a weather station (or a branch station) will first be established to obtain reliable measures of these weather parameters.
- The staff of the village weather station will regularly monitor the weather parameters.

Damage assessment based verification:

- Another option is based on the assessment of actual damage on your farm by an expert. It does not require any measurement of weather parameters. Hence, danger level is not relevant for this insurance scheme.
- An independent assessment of your crop damage will be made by an expert after your chosen hazard has taken place.
- There is a maximum limit of compensation that you can receive.
- However, your actual compensation can be less than this maximum amount. For example, assume that you buy an insurance scheme for which maximum compensation of your crop damage is Tk 5,000. After hazard strikes, the insurance provider verifies your crop damage and finds that your actual damage is Tk 2,000 per 33 decimal of your land. In that case, you will receive Tk 2,000 as compensation. But if your actual damage is assessed to be Tk 6,000 per 33 decimal of your land, you will still receive Tk 5000.

Do you understand the different between weather index based and damage verification based insurance schemes?

1 = no>>((Enumerator: please explain again)

2 = yes

Remember:

- Both insurance schemes will be applicable only for the weather related hazard that you choose. For example, imagine you buy an inundation insurance scheme, but your crop is damaged by hailstorm. In this case you will not receive any compensation.
- You can buy these insurance schemes only for maize cultivation during *rabi* season.

Provider:

- Insurance provider is part of these insurance schemes.
- You will make your decision to buy insurance based on your favorite insurance provider.

Availability:

- The insurance will be available in your village in the future only if we identify sufficient demand for it.
- Also, the cost of offering these contracts needs to be compared with the potential income they are likely to generate for the insurance provider.
- At the moment, we do not know the date when the insurance scheme might become available.

Next, I will show you FOUR cards. Each card will present two insurance options.

The options will vary based on:

- - Hazard type (inundation, hailstorm, windstorm)
  - The amount of deposit you have to make on December 15 (the 1^st^ day of *Poush*)
  - The amount of money you will receive on June 15 (the 1^st^ day of *Ashar*) if your chosen hazard takes place
  - The amount of money you will receive on June 15 (the 1^st^ day of *Ashar*) if your chosen hazard does NOT take place
  - Verification process (weather index based vs. damage assessment based)
  - Insurance provider option (Government bank, NGOs, private bank, private insurance company, Islamic bank).

**Appendix 6.** Description of the sampled farmer households and respondents.

| **Variable** | **Descriptive statistics** |
| --- | --- |
| ***Respondent characteristics*** |  |
| Male (%) | 60 |
| Average age (in years) (min-max) | 41 (20‒70) |
| High school and above (%) | 29 |
| No familiarity with insurance (%) | 63 |
| Risk aversion coefficient^a^ (θ) | 0.86 |
| Time preference^b^ (% with time discount rate > 70%) | 88 |
| ***Household characteristics*** |  |
| Religion (non-Muslim) (%) | 5% |
| Average household size (mean) | 6.25 |
| Size of cultivable land in decimal (40.4g m^2^) and hectare (mean) | 63 (0.25) |
| Value of non-land asset (in USD) (mean) | 1,608 |
| Size of maize cropped area in decimal (40.4g m^2^) and hectare (mean) | 26.42 (0.11) |
| Per capita monthly household expenditure (food + non-food) (USD) | 28 |
| Household below poverty line (%)^c^ | 38 |
| Savings (formal) account (%) | 40 |
| Credit (formal) account (%) | 48 |
| Purchased insurance (%) | 19 |

Notes:

^a^Assuming constant relative risk aversion (CRRA), $u\left( y \right)=\frac{y^{1-\theta}}{1-\theta}$, the curvature of the utility function θ represents the degree of risk aversion. This was determined by calculating the value of θ that would make a respondent indifferent between the chosen gamble and the two adjacent gambles (Eckel and Grossman, 2008).

^b^ Discount rate is determined solving the value function $v\left( M_{0} \right)=\frac{1}{1+r}v(M_{t})$. *M*_0_ is the present value of *M_t_* offered at time *t* with discount rate *r*.

^c^The expenditure data were used to estimate head count poverty rates following the ‘upper’ poverty line expenditure data released by the Bangladesh Bureau of Statistics (BBS 2011). The ‘upper’ expenditure comprises the values of both food and non-food items needed to ensure minimum subsistence which was estimated at Tk 1311 (USD17) per capita per month in 2010. Note that the national rural ‘upper’ poverty rate in Bangladesh is 35% (BBS 2011).

**Appendix 7.** Degree of climate change skepticism as reported by the survey respondents (*n =* 120).

| Question |  | Response | Percent of respondents |
| --- | --- | --- | --- |
| Have you observed any change in your local climate over the past 20 years? |  | Yes | 85 |
|  |  | No | 15 |
| Do you believe that climate change is caused by harmful pollution emitted by developed countries? |  | Yes | 85 |
|  |  | No | 11 |
|  |  | I don’t know | 4 |
| Do you worry or are you concerned about the harmful impacts of climate change on your lives and livelihoods? |  | I am very worried | 76 |
|  |  | No, I am not worried at all | 24 |

**Appendix 8.** Farmers’ perceived probability of experiencing natural hazards affecting their maize crop in the future (percent of farmer respondents, *n* = 120)

|  | “I don’t know - Only God knows” | Once every one year | Once every two years | Once every three or more years | Total |
| --- | --- | --- | --- | --- | --- |
| Windstorm | 52 | 40 | 6 | 2 | 100 |
| Inundation / waterlogging | 62 | 14 | 18 | 3 | 100 |
| Hailstorm | 64 | 21 | 7.5 | 7.5 | 100 |

**Appendix 9.** Marginal willingness to pay estimates (or mean implicit prices) for weather insurance in USD/season/bigha^a^ (95% confidence interval^b^ in the parenthesis).

| Attributes | Description | Marginal willingness to pay (MWTP) |
| --- | --- | --- |
| *Bad Time Payment* | MWTP for US$13 (Taka 1,000) worth of remuneration as compensation for a *standalone* *hailstorm* based *WII* contract | 2.80  (1.1−4.52) |
| *Good Time Payment* | MWTP for US$13 (Taka 1,000) worth of savings returned for a *hailstorm* based *WII* contract in case of no hazard event | 9.32  (7.50−11.14) |
| *Standard* | MWTP for standard insurance compared to WII | 6.52  (3.00−10.10) |
| *Private* | MWTP for private insurance providers (i.e. private bank and private insurance company) compared to other providers (i.e. government bank, NGOs and Islamic organizations) | -6.64  ((-11.70) −(-1.60)) |
| *Inundation* | MWTP for inundation compared to hailstorm WII | 0.83  (-3.24−4.90) |
| *Wind* | MWTP for windstorm compared to hailstorm WII | 1.80  (-2.33−6.00) |

Notes:

^a^One *bigha* = 0.134 ha.= 33 decimals (at 40.46m^2^ decimal^–1^)

^b^Confidence intervals were estimated using the Wald procedure (Delta Method).
